# Diagnosis of Trombiculosis by Videodermatoscopy

**DOI:** 10.3201/eid2006.130767

**Published:** 2014-06

**Authors:** Maria R. Nasca, Francesco Lacarrubba, Giuseppe Micali

**Affiliations:** University of Catania, Catania, Italy

**Keywords:** trombiculosis, trombiculiasis, Neotrombicula autumnalis, ectoparasitoses, skin infestation, dermoscopy, dermatoscopy, videodermatoscopy, epiluminescence, entodermoscopy, parasites

**To the Editor:** Dermoscopy (also known as dermatoscopy, epiluminescence microscopy, and surface microscopy) is a noninvasive technique that enables rapid and magnified (×10) in vivo observation of the skin and detection of morphologic details often not visible to the naked eye. Videodermatoscopy, which is performed with a probe equipped with lenses providing higher magnification (up to ×1,000) and connected to a personal computer, enables more detailed inspection of the skin than does manual dermoscopy and enables storage of digital images. Both techniques have been widely used for the differential diagnosis and monitoring of pigmented lesions; however, a role for these techniques in the diagnosis and follow-up of other skin disorders has recently emerged (*1*,*2*). Their usefulness for diagnosing several parasitic disorders of the skin (e.g., scabies, pediculosis, phthiriasis, larva migrans, tungiasis, myiasis, and tick infestations) has led to introduction of the term entodermoscopy. In the hands of trained physicians, these techniques are more effective than traditional methods (e.g., parasite identification by microscopic examination of samples obtained by skin scraping); they are well accepted by patients and particularly suitable for mass screening and posttreatment follow-up examinations (*1*–*8*).

We describe a puzzling case in which videodermatoscopy enabled a definitive diagnosis of trombiculosis. Trombiculosis is a common but underreported ectoparasitosis that is probably often misdiagnosed.

In January 2013, a 66-year-old man from eastern Sicily, Italy, reported diffuse intense pruritus that persisted despite various treatments administered in the previous months for a well-documented diagnosis of scabies. The condition had considerably impaired his quality of life, causing family concerns and missed workdays. Physical examination revealed multiple excoriations and pinpoint erythematous macules scattered throughout the trunk and lower legs (Figure, panel A), but no burrows or other findings suggestive of scabies were detectable with use of a common magnification lens. An accurate and thorough examination by videodermatoscopy (at ×150 magnification) revealed a reddish mite strongly attached to the skin on the patient’s right shin. In the stored images, a larval *Neotrombicula autumnalis* mite was subsequently identified (Figure, panel B). A diagnosis of cutaneous trombiculosis was made, and the patient was instructed to avoid further environmental exposure; his symptoms were consequently relieved.

**Figure:**
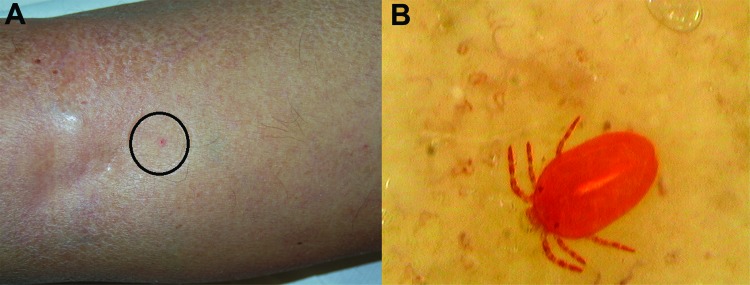
Clinical features of a nonspecific lesion (A) and its corresponding, unequivocal dermoscopy findings (B), showing a *Neotrombicula autumnalis* mite attached to the skin (magnification ×150).

Trombiculosis is an infestation of the skin by the larval stage of various species of mites belonging to the phylum Arthropoda, class Arachnida, subclass Acarina. *N. autumnalis* mites are more diffuse in the temperate and humid European environment, where adult individuals live and reproduce on the soil, especially during warmer and wet late summer months. Eggs usually hatch at the end of autumn, and new mites, which at their larval stage are obligate parasites of warm-blooded hosts, usually feed and grow on the skin of small rodents and dogs, injecting lytic enzymes to digest cutaneous cells. Humans engaged in outdoor activities or staying in the countryside for professional or recreational purposes can become occasional hosts of this ectoparasite. Infection is more common in autumn and should be suspected for persons at risk (e.g., farmers, hunters, children) who have an itchy eruption with a likely environmental cause (*9*).

No specific medications are required to treat trombiculosis in humans. Usually effective measures are use of repellents, avoidance of exposure by wearing adequate clothing when in mite-infested areas, and washing of body and clothes with soap and hot water immediately after exposure. Itch can sometimes be relieved by supportive care with oral antihistamines or topical corticosteroids (*9*). Antimicrobial drugs might be needed to cure bacterial superinfection resulting from repeated scratching.

Trombiculosis is not considered rare, but it is underreported and, probably, often misdiagnosed. Cutaneous findings are nonspecific, and an accurate anamnesis is essential for making this challenging diagnosis. Because the patient reported here denied any professional or recreational outdoor activities, a single clinical examination would probably have led to a wrong diagnosis of a nonspecific itchy dermatitis, leading to use of inadequate or needless medications. Also, our experience confirms that common magnification lenses and even dermoscopy at ×10 magnification have some limitations; parasites can easily be missed or barely noticeable so that their identification can be quite difficult. In such instances, videodermatoscopy might lead to the diagnosis and should be considered as a useful diagnostic aid. Image storage and sharing can also facilitate collaboration with experts and can enable timely recognition of unusual parasitic disorders imported from different geographic areas or tropical countries. 

The cost of the equipment varies according to resolution quality, magnification capability, and image storage facility; costs range from 500 (for simple systems) to 10,000 (for sophisticated systems) euros. The expense is greatly outweighed by the advantages of avoiding the high cost of managing outbreaks of epidemic parasitoses resulting from misdiagnosis, treatment failures, and incomplete posttreatment monitoring (*10*). 

Videodermatoscopy is a noninvasive way to diagnose some pruritic disorders while avoiding unnecessary, uncomfortable, and sometimes expensive investigations and treatments. Physicians without access to such equipment should consider promptly referring patients to the nearest available videodermatoscopy service for effective management.
